# Risk factors of paclitaxel-induced peripheral neuropathy in patients with breast cancer: a prospective cohort study

**DOI:** 10.3389/fonc.2024.1327318

**Published:** 2024-03-07

**Authors:** Sun Lixian, Yu Xiaoqian, Guo Luyan, Zhou Lizhi, Du Rui, Yao Hongyue, Zhao Caijie, Yuan Fenghui

**Affiliations:** ^1^ School of Nursing and Rehabilitation, North China University of Science and Technology, Tangshan, China; ^2^ Breast Surgery, Tangshan People’s Hospital, Tangshan, China; ^3^ School of Nursing, Tangshan Vocational and Technical College, Tangshan, China; ^4^ Department of Neurology, Kailuan General Hospital, Tangshan, China

**Keywords:** paclitaxel, peripheral neuropathy, breast cancer, chemotherapy, risk factor

## Abstract

**Objective:**

Chemotherapy-induced peripheral neuropathy (CIPN) is a common and severe adverse reaction in taxane-based chemotherapy. This study aimed to analyze the risk factors of peripheral neuropathy in patients with breast cancer receiving paclitaxel chemotherapy to provide a reference for the early prevention of CIPN.

**Methods:**

We included 350 patients with breast cancer who received chemotherapy for the first time at the Tangshan People’s Hospital between August 2022 and June 2023 and were followed for at least 3 months after the end of chemotherapy. The incidence of CIPN in patients with breast cancer was calculated, and risk factors for CIPN were analyzed using logistic regression analysis.

**Results:**

The incidence rate of CIPN was 79.1%. Multifactor logistic regression analysis indicated that age ≥45 years [odds ratio (OR)=5.119, 95% confidence interval (CI)=1.395–18.780] and ≥60 years (OR=9.366, 95% CI=1.228–71.421), history of hypertension (OR=3.475, 95% CI=1.073–11.250), cumulative dose of chemotherapy drugs >900 mg (OR=4.842, 95% CI=1.961–5.946), vitamin D deficiency (OR=6.214, 95% CI=2.308–16.729), abnormal alanine aminotransferase (OR=3.154, 95% CI=1.010–9.844), anemia before chemotherapy (OR=2.770, 95% CI=1.093–7.019), infusion duration of chemotherapy drugs >30 min (OR=3.673, 95% CI=1.414–9.539), body mass index ≥24 kg/m^2^ (OR=8.139, 95% CI=1.157–57.240), mild depression (OR=4.546, 95% CI=1.358–15.223), and major depression (OR=4.455, 95% CI=1.237–16.037) increased the risk of CIPN. Having a regular caregiver (OR=0.223, 95% CI=0.087–0.573), high levels of physical activity (OR=0.071, 95% CI=0.008–0.647), and strong social support (OR=0.048, 95% CI=0.003–0.682) were protective factors against CIPN.

**Conclusion:**

Clinical attention should be paid to patients with these risk factors, and active and effective preventive measures should be taken to reduce the occurrence of CIPN and improve the quality of life.

## 1 Introduction

Adjuvant chemotherapy is frequently needed for patients with breast cancer, and chemotherapy can improve the survival rate while reducing the risk of recurrence and metastasis. However, chemotherapy-associated toxicities have a negative impact on the patient recovery. Chemotherapy-induced peripheral neuropathy (CIPN) is a common and serious adverse effect of chemotherapy with paclitaxel drugs. It is mainly characterized by sensory abnormalities, including numbness, tingling, burning pain, and increased sensitivity, in the distal limbs in a “sock-glove” distribution (1, 2). CIPN interferes with the daily life and patient activities, such as typing, writing, and buttoning up clothes, and increases the risk of falls and injuries. It frequently occurs during the period of chemotherapy; the incidence may reach 68.1–84%, and >30% of patients may experience chronic peripheral neuropathy. Patients experience symptoms for a long period (3), which seriously affects their functional status and quality of life and ultimately influences their survival. However, there is currently, no effective treatment for CIPN (4). Therefore, identifying effective predictive factors for the occurrence of CIPN has received increasing attention. Particularly, exploring risk factors for CIPN has become a hot research topic owing to the lack of predictors of CIPN (5). Known risk factors include cumulative dose (6), age ≥45 years (7), and body mass index (8); however, other factors, such as the duration of the patient’s chemotherapeutic drug infusion (9), functional activity (10), hepatic function, anemia (11), patient’s debilitating condition (12), and psycho-psychological factors (13), among many other potential risk factors, have yet to be adequately investigated. The National Cancer Institute Toxicity Scoring Criteria is currently the most frequently used clinical tool to diagnose CIPN (14). This study aimed to analyze the risk factors for CIPN in patients with breast cancer receiving paclitaxel chemotherapy to provide guidance for healthcare professionals to develop targeted preventive measures.

## 2 Materials and methods

### 2.1 Study design and population

This prospective cohort study was approved by the Ethics Committee of Tangshan People’s Hospital (RMYY-LLKS-2022-055) and was conducted according to the tenets of the Declaration of Helsinki. Participation was voluntary, and informed consent was obtained from all patients.

**Figure 1:**
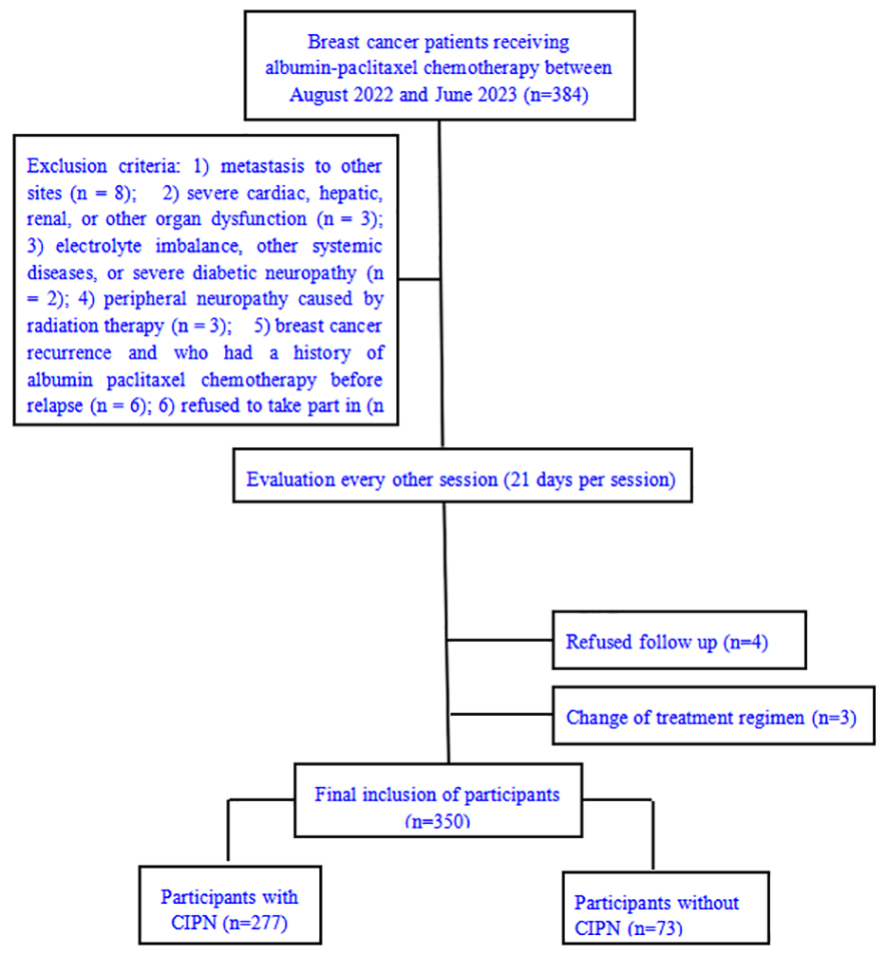
Patient inclusion flowchart.

The study employed an 80% certainty level based on previous studies, and calculated using the PASS software (NCSS Corp, Kaysville, UT, USA), with a target sample size of 320 patients. Patients with breast cancer treated with paclitaxel between August 2022 and June 2023 at Tangshan People’s Hospital were included in this study (**Figure 1**). The inclusion criteria were as follows: 1) age ≥18 years; 2) breast cancer diagnosis confirmed through pathological examination; 3) treatment with taxane-based chemotherapeutic drugs; 4) peripheral neuropathy grade 0 according to the National Cancer Institute Common Terminology Criteria for Adverse Events; 5) absence of psychiatric disorders or communication barriers; 6) normal hearing; 7) sufficient reading, expression, and communication abilities; and 8) ability to cooperate in completing the questionnaire surveys. The exclusion criteria were as follows: 1) metastasis to other sites; 2) severe cardiac, hepatic, renal, or other organ dysfunction; 3) electrolyte imbalance, other systemic diseases, or severe diabetic neuropathy; 4) peripheral neuropathy caused by intoxication, infection, chemotherapy, radiation therapy, or other factors; and 5) a history of breast cancer recurrence and chemotherapy treatment before recurrence. Finally, 350 patients were included.

### 2.2 Study protocol

This study used the time of the patient’s first chemotherapy session as the starting point for follow-up. Follow-up assessments were performed after each chemotherapy cycle. The study endpoint was the occurrence of CIPN events or the end of the follow-up period if no CIPN occurred. During this period, basic and relevant data on patients who had CIPN were recorded. General patient information was collected through face-to-face interviews, and relevant laboratory indicators were reviewed in the electronic medical record system.

The general demographic characteristics questionnaire was designed by the researchers. Data on general demographic characteristics, daily behavior habits, past medical history, and related laboratory indicators of the patients were collected. CIPN was evaluated according to international standards using the National Cancer Institute Common Terminology Criteria for Adverse Events (14), as follows: grade 0: normal findings; grade 1: loss of tendon reflex or abnormal sensation (including paresthesia); grade 2: altered or abnormal sensations (including paresthesia) affecting limb function without impacting daily activities; grade 3: altered or abnormal sensations (including paresthesia) affecting daily activities; grade 4: life-threatening consequences requiring urgent intervention; and grade 5: death.

Psychological evaluation was performed using the Hospital Anxiety and Depression Scale (15). Briefly, this tool is composed of 14 entries, including 7 anxiety self-assessment scales and 7 depression self-assessment scales. The scores of the two subscales of anxiety and depression are added, and patients with scores of 0–7 are classified as asymptomatic; 8–10, symptomatic and suspicious; and 11–21, definitely symptomatic. Sleep quality was evaluated using the Pittsburgh Sleep Quality Index Scale (16). Entries were categorized into the following seven dimensions: subjective sleep quality, time to sleep, sleep duration, sleep efficiency, sleep disorders, hypnotic medications, and daytime functioning. Each component was scored 0–3, and the cumulative score of each component was the total Pittsburgh Sleep Quality Index Scale score. The scores ranged from 0–21, with higher scores indicating poorer sleep quality. Social support was evaluated using the Social Support Rating Scale (17). Briefly, this scale comprises the following three dimensions: objective support (three items), subjective support (four items), and support utilization (three items). The total score is 12–66, with higher scores indicating higher levels of social support. Physical activity was evaluated using the International Physical Activity Questionnaire (18). Specifically, the International Physical Activity Questionnaire - Short Form, which included three sections of walking, moderate physical activity, and heavy physical activity and a total of seven questions, was used. Total energy values were calculated based on the metabolic equivalent corresponding to each type of physical activity.

### 2.3 Statical analysis

Statistical analysis was performed using IBM SPSS Statistics for Windows, version 26.0 (IBM Corp, Armonk, N.Y, USA). Categorical data are described using frequency counts and percentages. Single-factor analysis was conducted using the chi-square test and single-factor logistic regression analysis. Multifactor analysis was performed using binary logistic regression analysis. Variables that exhibited statistical significance in the single-factor analysis were included in the multifactor binary logistic regression analysis. The odds ratios (ORs) and 95% confidence intervals were calculated for each factor to identify the risk factors. The significance level for testing was set at a two-tailed α=0.05.

## 3 Results

### 3.1 Univariate analysis of CIPN in patients with breast cancer

All patients were female; 273 patients had CIPN, and 77 patients did not have CIPN, with a prevalence rate of 79.1%. The patients’ age ranged from 26 years to 75 years, with a mean of 52.44 ± 10.252 years. Overall, 30.3%, 43.7%, and 26.0% of the patients were aged 18–44, 45–59, and ≥60 years, respectively, and 47.1% were not menopausal, and 52.9% were menopausal. Regarding education, 22.9% of the patients had an education level of elementary school or below; 40.9%, junior high school; 24.9%, senior high school or middle school; and 11.4%, college or above. Additionally, 11.4% of the patients were married. Regarding marital status, 90.3%, 6.3%, and 3.4% of the patients were married, divorced, and widowed, respectively. The molecular types were human epidermal growth factor receptor 2+ in 35.1%; luminal A in 28.6%; luminal B in 28.6%, and triple negative in 7.7%. The cumulative chemotherapeutic drug dose was ≤900 mg and >900 mg in 46.6% and 53.4%, respectively.

Single-factor analysis revealed statistically significant differences in age, menopausal status, body mass index, body surface area, duration of chemotherapy infusion, daily water intake after chemotherapy, presence of a caregiver, hypertension, diabetes, tumor node metastasis stage, cumulative chemotherapy dose, vitamin D deficiency, abnormal alanine transaminase levels, abnormal aspartate transaminase levels, glucose abnormalities, pre-chemotherapy anemia, physical activity level, anxiety, depression, social support, and sleep quality (all P<0.05) between the patients with and without CIPN. These were potential risk factors for CIPN development. However, education level, marital status, occupational status, smoking, alcohol consumption, heart disease, mode of surgery, white blood cell count, debilitating lymphocyte count, and healthy lifestyle were not significant (P >0.05) and were not associated with the occurrence of CIPN. Further details are presented in **Table 1**.

**Table 1:** Univariate analysis of significant factors of peripheral neuropathy in patients with and without CIPN (n=350).

Characteristic	CIPN, n (%)	No CIPN, n (%)	*X^2^*	P	OR	95% CI
Age						
18–45 years	64 (23.1)	42 (57.5)	37.008	<0.001	Ref	
46–59 years	127 (45.8)	26 (35.6)	15.821	<0.001	3.206	1.806–5.691
≥60 years	86 (31.0)	5 (6.8)	23.398	<0.001	11.287	4.228–30.137
Menopause						
No	116 (41.9)	49 (67.1)			Ref	
Yes	161 (58.1)	24 (32.9)	14.778	<0.001	2.834	1.645–4.880
Education						
High school diploma or less	250 (90.3)	60 (82.2)	1.967	0.161	0.548	0.236–1.270
College degree or more	27 (9.8)	13 (17.8)	6.468	0.011	0.297	0.116–0.757
Marital status						
Married	251 (90.6)	65 (89)	0.692	0.707	Ref	
Divorced	16 (5.8)	6 (8.2)	0.552	0.458	0.691	0.260–1.835
Widowed	10 (3.6)	2 (2.7)	0.108	0.743	1.295	0.277–6.055
Employment						
Peasantry	84 (30.3)	28 (38.4)	7.363	0.051	Ref	
Housewife	91 (32.9)	24 (32.9)	5.620	0.018	2.792	1.195–6.524
Retired	102 (36.8)	21 (28.8)	0.988	0.320	1.412	0.715–2.786
History of smoking						
No	274 (98.9)	71 (97.2)			Ref	
Yes	3 (0.01)	2 (2.7)	0.797	0.372	1.011	0.999–1.023
Alcohol consumption						
No	273 (98.6)	71 (97.2)			Ref	
Yes	4 (1.4)	2 (2.7)	0.576	0.448	0.520	0.093–2.897
BMI						
<24 kg/m^2^	111 (40.0)	49 (67.1)			Ref	
≥24 kg/m^2^	166 (60.0)	24 (32.9)	17.036	<0.001	3.053	1.772–5.262
BSA						
≤1.6 m^2^	141 (50.9)	47 (64.4)			Ref	
>1.6 m^2^	136 (49.1)	26 (35.6)	4.224	0.040	1.744	1.022–2.974
Duration of chemotherapy infusion						
≤30 min	135 (48.7)	52 (71.2)			Ref	
>30 min	142 (51.3)	21 (28.8)	11.751	0.001	2.605	1.489–4.555
Water intake on the day after chemotherapy						
<1 L	168 (60.6)	32 (43.8)	6.829	0.033	Ref	
1–2 L	71 (25.6)	28 (38.4)	6.088	0.014	0.483	0.271–0.861
>2 L	38 (13.7)	13 (17.8)	2.442	0.118	0.557	0.267–1.161
Regular caregivers						
No	12 8(46.2)	19 (26.0)			Ref	
Yes	149 (53.8)	54 (74.0)	9.660	0.002	0.410	0.231–0.727
High blood pressure						
No	145 (52.3)	61(83.6)			Ref	
Yes	132 (47.7)	12 (16.4)	23.247	<0.001	4.628	2.386–8.974
Diabetes						
No	183 (66.1)	60 (82.2)			Ref	
Yes	94 (34.0)	13 (17.9)	7.079	0.008	2.371	1.239–4.537
Heart disease						
No	266 (96.0)	71 (97.3)			Ref	
Yes	11 (4.0)	2 (2.7)	0.245	0.621	1.468	0.318–6.774
Disease stage						
I	40 (14.4)	13 (17.9)	7.266	0.026	Ref	
II	117 (42.2)	41 (56.2)	0.042	0.837	0.927	0.452–1.905
III	120 (43.3)	19 (26.0)	3.157	0.075	2.053	0.931–4.527
Operation						
Breast-conserving surgery	91 (32.9)	19 (26.0)	2.717	0.437	Ref	
Modified radical operation	77 (27.8)	27 (37.1)	2.365	0.124	0.594	0.308–1.153
Mastectomy	103 (37.2)	25 (34.2)	0.200	0.655	0.860	0.445–1.664
Other (prosthesis)	6 (2.1)	2 (2.7)	0.300	0.584	0.626	0.117–3.344
Cumulative dose of chemotherapy drugs						
≤900 mg	112 (40.4)	51 (70.0)			Ref	
>900 mg	165 (59.6)	22 (30.1)	20.110	<0.001	3.415	1.961–5.946
Vitamin D deficiency						
No	73 (26.4)	32 (43.8)			Ref	
Yes	204 (73.7)	41 (56.2)	8.408	0.004	2.181	1.279–3.721
Glutamic-pyruvic transaminase						
Normal	106 (38.3)	61 (83.6)			Ref	
Abnormal	171 (61.7)	12 (16.4)	47.511	<0.001	8.200	4.219–15.914
Glutamic oxaloacetic transaminase						
Normal	170 (61.4)	60 (82.2)			Ref	
Abnormal	107 (38.6)	13 (17.8)	11.115	0.001	2.905	1.522–5.450
Blood glucose						
Normal	105 (37.9)	39 (53.4)			Ref	
Abnormal	172 (62.1)	34 (46.6)	5.746	0.017	1.879	1.117–3.161
White blood cell count						
Normal	231 (83.4)	59 (81.0)			Ref	
Abnormal	46 (16.6)	14 (19.2)	0.269	0.604	0.839	0.432–1.629
Lymphocyte count						
Normal	222 (80.1)	58 (79.4)			Ref	
Abnormal	55 (19.9)	15 (20.5)	0.017	0.895	1.044	0.550–1.980
Anemia before chemotherapy						
No	107 (38.6)	60 (82.2)			Ref	
Yes	170 (61.4)	13 (17.8)	43.949	<0.001	7.333	3.841–13.998
Asthenia						
No asthenia	109 (39.4)	24 (32.9)	1.957	0.376	Ref	
Pre-asthenia	134 (48.4)	42 (57.5)	1.519	0.218	0.702	0.401–1.232
Asthenia	34 (12.3)	7 (9.6)	0.020	0.887	1.069	0.424–2.699
Physical activity status						
Low grade	67 (24.2)	2 (2.7)	30.877	<0.001	Ref	
Middle grade	177 (63.9)	46 (63.0)	8.635	0.003	0.115	0.027–0.486
High grade	33 (11.9)	25 (34.2)	17.870	<0.001	0.039	0.009–0.176
Anxiety						
No anxiety	95 (34.3)	39 (53.4)	8.950	0.011	Ref	
Mild anxiety	108 (39.0)	20 (27.4)	6.641	0.010	2.217	1.210–4.061
Severe anxiety	74 (26.7)	14 (19.2)	4.955	0.026	2.170	1.097–4.292
Depression						
Not depressed	95 (34.3)	44 (60.3)	16.286	<0.001	Ref	
Mild depression	101 (36.5)	16 (22.0)	10.894	0.001	2.924	1.546–5.529
Severe depression	81 (29.2)	13 (17.8)	9.167	0.002	2.886	1.453–5.731
Social support						
Low	46 (16.6)	1 (1.4)	36.273	<0.001	Ref	
Moderate	182 (65.7)	36 (49.3)	4.622	0.032	0.110	0.015–0.823
High	49 (16.7)	36 (49.3)	11.583	0.001	0.033	0.004–0.225
Sleep quality						
Poor	72 (26.0)	8 (11.0)	7.412	0.025	Ref	
Ordinary	102 (36.8)	32 (43.8)	5.988	0.014	0.354	0.154–0.813
Good	103 (37.2)	33 (45.2)	6.269	0.012	0.347	0.151–0.795
Healthy lifestyle						
Low	14 (5.1)	4 (5.5)	1.431	0.489	Ref	
Moderate	241 (87.0)	60 (82.2)	0.055	0.814	1.148	0.365–3.612
High	22 (7.9)	9 (12.3)	0.270	0.604	0.698	0.180–2.708

The chi-square test is used, and P<0.05 is considered statistically significant.

CIPN, chemotherapy-induced peripheral neuropathy; OR, odds ratio; CI, confidence interval; BMI, body mass index; BSA, body surface area; Ref, reference.

### 3.2 Binary logistic multivariate analysis of CIPN in patients with breast cancer

Using the presence of peripheral neuropathy as the dependent variable, the variables with statistical significance in the univariate analysis were set as independent variables in the multivariate analysis. Multivariate logistic regression analysis revealed that age 45–59 years (OR=5.119) and ≥60 years (OR=9.366), history of hypertension (OR=3.475), cumulative dose of chemotherapy drugs >400 mg (OR=4.842), vitamin D deficiency (OR=6.214), alanine transaminase abnormality (OR=3.154), pre-chemotherapy anemia (OR=2.770), chemotherapy drug infusion duration >30 min (OR=3.673), body mass index ≥24 kg/m^2^ (OR=8.139), mild depression (OR=4.546), and severe depression (OR=4.455) were risk factors for CIPN in patients with breast cancer. Meanwhile, having a caregiver (OR=0.002), higher levels of physical activity (OR=0.071), and higher levels of social support (OR=0.048) were protective factors. Further details are presented in **Table 2**. Risk factors are those that cause CIPN or increase the probability of its occurrence; protective factors are those that prevent or minimize the occurrence of CIPN.

**Table 2:** Multivariate analysis of independent predictive factors of the development of CIPN (n=350).

Characteristics	Wald	P	OR	95% CI
Age				
45–59 years vs. 18–44 years	6.064	0.014	5.119	1.395–18.780
≥60 years vs. 18–44 years	4.658	0.031	9.366	1.228–71.421
Disease stage				
II vs. I	0.863	0.353	0.559	0.164–1.906
III vs. I	0.011	0.915	0.078	0.273–4.259
Menopause				
Yes vs. no	3.497	0.061	0.297	0.083–1.060
Regular caregivers				
Yes vs. no	9.703	0.002	0.223	0.087–0.573
High blood pressure				
Yes vs. no	4.319	0.038	3.475	1.073–11.250
Diabetes				
Yes vs. no	1.231	0.267	0.508	0.153–1.682
Cumulative dose of chemotherapy drugs				
>900 mg vs. <900 mg	8.748	0.003	4.842	1.702–13.770
Vitamin D deficiency				
Yes vs. no	13.071	<0.001	6.214	2.308–16.729
Glutamic-pyruvic transaminase				
Abnormal vs. normal	3.912	0.048	3.154	1.010–9.844
Blood glucose				
Abnormal vs. normal	0.286	0.593	0.782	0.318–1.926
Glutamic oxalacetic transaminase				
Abnormal vs. normal	0.142	0.706	1.240	0.405–3.796
Anemia before chemotherapy				
Yes vs. no	4.613	0.032	2.770	1.093–7.019
Physical activity status				
Moderate vs. low	1.459	0.227	0.311	0.047–2.071
High vs. low	5.507	0.019	0.071	0.008–0.647
Duration of chemotherapy infusion				
>30 min vs. ≤30 min	7.140	0.008	3.673	1.414–9.539
Water intake on the day after chemotherapy				
1–2 L vs. <1 L	0.235	0.628	0.801	0.327–1.965
>2 L vs. <1 L	0.471	0.492	0.656	0.197–2.185
BMI				
≥24 kg/m^2^ vs. <24 kg/m^2^	4.438	0.035	8.139	1.157-57.240
BSA				
>1.6 m^2^ vs. 1.6 m^2^	1.581	0.209	0.286	0.041–2.012
Anxiety				
Mild vs. None	0.060	0.807	1.143	0.391–3.341
Severe vs. None	0.571	0.450	0.621	0.181–2.135
Depression				
Mild vs. None	6.031	0.014	4.546	1.358–15.223
Severe vs. None	5.226	0.022	4.455	1.237–16.037
Social support grade				
Middle vs. Low	1.522	0.217	0.198	0.015–2.593
High vs. Low	5.030	0.025	0.048	0.003–0.682
Sleep quality				
Ordinary vs. poor	0.509	0.476	0.614	0.160–2.237
Good vs. poor	0.048	0.826	1.169	0.291–4.693

CIPN, chemotherapy-induced peripheral neuropathy; OR, odds ratio; CI, confidence interval; BMI, body mass index; BSA, body surface area

Using binary logistic regression test, P<0.05 is considered statistically significant.

## 4 Discussion

This study found a significant correlation between age and CIPN, with older patients (≥45 years) being more likely to experience neurotoxicity during chemotherapy. This is consistent with the results of Pabst et al. (19). This may be due to the gradual decline in organ function with age. Older adults may have worse circulation and metabolism than younger adults. Additionally, decreased cellular repair capacity and neurotrophic deficits may account for the higher susceptibility and severe CIPN in older adults (20). Therefore, when dealing with peripheral neuropathy in patients with breast cancer, medical staff should pay more attention to elderly patients. Furthermore, more research is needed on the risk and protective factors of CIPN in older adults to prevent and treat CIPN more effectively.

This study found that hypertension is a risk factor for CIPN in patients with breast cancer. Long-term hypertension can lead to chronic peripheral vascular damage or peripheral tissue changes, and the increase in blood pressure can lead to the damage of vascular pressure sensor function, resulting in the decline of autonomic nervous regulation function in patients, and the two form a vicious circle to aggravate autonomic neuropathy (21). Therefore, hypertension can damage the autonomic nerve function and subsequently aggravate peripheral neuropathy. Therefore, it is crucial to pay attention to blood pressure control and implement corresponding preventive measures in patients with breast cancer who have hypertension.

In general, the required chemotherapy dose increases with the patient’s body mass index. This increases the incidence and severity of CIPN. Furthermore, the relationship between body mass index and the nervous system is complex. Obese patients frequently have underlying conditions, such as type 2 diabetes, hypertension, and osteoarthritis. Chemotherapy with taxanes (e.g., paclitaxel) can exacerbate peripheral neuropathy in patients with diabetes. Additionally, animal model studies have shown that obesity can lead to microvascular damage and peripheral nervous system dysfunction (20). Moreover, studies have suggested that obesity is a risk factor for other neuropathies, such as oxaliplatin-induced peripheral neuropathy (22) and diabetic neuropathic conditions (23). In this context, to protect the health of patients, medical staff training on the assessment and management of CIPN symptoms should be increased, and early and correct assessment and intervention measures should be provided in patients with a high body mass index. Overweight patients should be advised to control their body weight, eat small meals, and engage in physical exercise.

This study found a correlation between vitamin D deficiency and CIPN. This may be because vitamin D potentially plays a role in axonal repair, making vitamin D-deficient patients more susceptible to severe CIPN. Vitamin D can induce the expression of nerve growth factor, which can promote the growth, maturation, and differentiation of neurons; maintain the normal physiological function of nerve cells; and repair damaged nerve cells. However, reductions in nerve growth factor can make nerve fibers vulnerable to damage by various factors (24, 25). These findings are consistent with the results of our analysis and provide a strong rationale for confirmatory studies on the association between pretreatment vitamin D deficiency and PN in large independent prospective cumulative patient cohorts receiving paclitaxel and other neurotoxic chemotherapeutic agents (26). Therefore, healthcare providers should advise patients to supplement vitamin D in a timely manner to prevent CIPN.

Chemotherapy-induced liver damage and pre-chemotherapy anemia are common during treatment in patients with cancer. Chemotherapeutic drugs can directly damage liver cells, leading to liver damage and elevated transaminase levels. They can also cause sinusoidal liver injury, leading to cholestatic hepatitis, elevated bilirubin levels, or a combination of the two. Given that most chemotherapeutic drugs are primarily metabolized and excreted by the liver, any impairment in liver function can alter the metabolism and secretion of these drugs, prolonging their action time and potentially increasing the incidence and severity of neuropathy. Although anemia is relatively common in patients with cancer, research on the relationship between anemia and CIPN is limited. In this study, patients with concomitant anemia were more likely to have CIPN. This may have been because of anemia-induced hypoxia in the peripheral tissues, as shown in previous studies. Peripheral tissue hypoxia can affect the function of the peripheral nervous system, and protein synthesis disturbances caused by tissue hypoxia can affect the repair of peripheral nerve damage (20). Clinicians should pay more attention to preventing neurotoxicity in patients with liver dysfunction and pre-chemotherapy anemia when administering chemotherapeutic drugs.

The results of this study showed that patients with breast cancer who received chemotherapy infusions for more than 30 min had a higher risk of developing CIPN. The possible reasons for this include drug metabolism and metabolite production. Rapid infusion can achieve steady-state drug concentrations faster, thereby reducing metabolite formation and potential side effects (27). Additionally, increasing the infusion time of the drug can prolong the time that paclitaxel remains in the body, resulting in a more extensive distribution of the drug in the extravascular and/or tissue compartments, thereby increasing the risk of CIPN. Therefore, clinicians should attempt to keep the infusion duration to within 30 min. We also found that depression and social support are risk factors for CIPN in patients with breast cancer, which aligns with the results of previous studies (4, 28, 29). Specifically, depression and social support can influence the occurrence of CIPN owing to the impact of physiological, psychological, and environmental factors on CIPN symptoms. Even when experiencing similar symptoms, individuals may present with diverse manifestations due to variations in their perceptions of physiological, psychological, and environmental factors (30). Social support provides patients with material and emotional assistance. It can also guide patients in communicating and interacting with others, using positive coping strategies to manage their symptoms, and developing an accurate understanding of CIPN. Moreover, high levels of social support can facilitate an early return to society and improve social functioning. Therefore, nurses should emphasize the positive impact of social support on CIPN symptoms. They should encourage patients to actively seek social support and engage in social activities, promote peer support and sharing, bolster patient confidence, enhance follow-up care, promptly identify issues, and provide guidance and assistance. Patients can be encouraged to help and understand each other through face-to-face, telephone, and Internet communication and other forms of mutual support and experience sharing. Related health education should also be provided to family members of patients, and follow-up work should be strengthened for vulnerable groups with low social support levels.

Our results showed that patients with breast cancer with a regular caregiver had a lower incidence of CIPN than those without a regular caregiver. This could be because patients with a regular caregiver usually have family members accompanying them during hospitalization (28). These caregivers had close contact with healthcare professionals and gained a better understanding of preventive nursing measures for CIPN. Therefore, they are better equipped to assist patients in preventing CIPN in their daily lives and improve the patient’s quality of life. Additionally, patients with a fixed caregiver can be accompanied to the toilet during the infusion of chemotherapy drugs; therefore, they are more likely to drink large amounts of water. This helps shorten the retention time of the drug, which may reduce the incidence and severity of neurotoxicity.

The results of this study showed that a low level of physical activity is an influential factor in the development of CIPN in patients with breast cancer. This is consistent with the findings of Srivastava et al. (31). Exercise has a positive effect on reducing CIPN symptoms in patients with cancer (32). This is because exercise prevents axonal degeneration, promotes axonal regeneration of injured peripheral nerves, reduces nociceptive hypersensitivity after peripheral nerve injury and promotes functional recovery, increases the expression of neurotrophic factors, improves the compensatory capacity of normal nerve fibers, and enhances the antioxidant activity of mitochondria (33). Our result suggests that in patients with breast cancer, CIPN symptoms can be managed through exercise interventions. Exercise intensity and duration should be tailored according to the physical activity level of chemotherapy patients, facilitating the establishment of a regular exercise routine and helping to prevent symptom deterioration.

This study had some limitations. First, it investigated only patients with breast cancer. Second, some patients used oral vitamin B6 for neurotrophic purposes during data collection, which may have affected the results of this study, although validity was not affected. Third, this was a cross-sectional study, and future studies should conduct a follow-up over a longer period. Finally, this study analyzed the correlation between influencing factors and CIPN to explore clinically significant risk factors for clinical reference. Future multicenter large-scale studies with longer follow-up observations are needed to explore the development of CIPN and provide a more scientific basis for the prevention and intervention of CIPN in patients receiving chemotherapy.

## 5 Conclusion

The primary risk factors for the occurrence of CIPN in patients with breast patients are age, history of hypertension, cumulative dose of chemotherapy drugs, vitamin D deficiency, abnormal liver function, pre-chemotherapy anemia, chemotherapy infusion duration >30 min, body mass index ≥24 kg/m^2^, depression, lack of a regular caregiver, low physical activity level, and low social support level. Therefore, when administering chemotherapy in clinical practice, close attention should be given to individuals with these factors, and proactive and effective preventive measures should be implemented to reduce the occurrence of peripheral neuropathy, improve quality of life, and prolong survival.

## Data availability statement

The original contributions presented in the study are included in the article/supplementary material. Further inquiries can be directed to the corresponding author.

## Ethics statement

The studies involving humans were approved by Ethics Committee of Tangshan People’s Hospital. The studies were conducted in accordance with the local legislation and institutional requirements. The participants provided their written informed consent to participate in this study. Written informed consent was obtained from the individual(s) for the publication of any potentially identifiable images or data included in this article.

## Author contributions

SL: Data curation, Formal analysis, Investigation, Methodology, Software, Validation, Visualization, Writing – original draft, Writing – review & editing. YX: Conceptualization, Data curation, Investigation, Software, Visualization, Writing – original draft. GL: Conceptualization, Data curation, Investigation, Software, Writing – original draft. ZL: Conceptualization, Funding acquisition, Methodology, Project administration, Resources, Supervision, Validation, Writing – review & editing. DR: Investigation, Resources, Supervision, Validation, Writing – review & editing. YH: Investigation, Supervision, Visualization, Writing – review & editing. ZC: Conceptualization, Methodology, Supervision, Writing – review & editing. YF: Formal analysis, Validation, Visualization, Writing – review & editing.
